# ‘The doctor doesn’t understand Xhosa and the service user doesn’t understand English’ - exploring the role of security guards acting as informal interpreters in psychiatric care in South Africa

**DOI:** 10.1186/s12913-024-11722-5

**Published:** 2024-10-16

**Authors:** Saskia Hanft-Robert, Lindokuhle Shongwe, Qhama Cossie, Philasande Sithole, Tessa Roos, Mike Mösko, Leslie Swartz

**Affiliations:** 1 Department for Medical Psychology, University Medical Center Hamburg-Eppendorf, Martinistraße 52, 20246 Hamburg, Germany; 2 Department of Psychology, Stellenbosch University, Stellenbosch, South Africa; 3 Department of Health & Wellness, Valkenberg Hospital, Cape Town, South Africa; 4 Department of Psychiatry and Mental Health, University of Cape Town, Cape Town, South Africa; 5 Department of Applied Human Sciences, Magdeburg-Stendal University of Applied Sciences, Magdeburg, Germany

**Keywords:** Interpreting, Security guards, Language Barrier, Mental Healthcare, Psychiatry, South Africa

## Abstract

**Introduction:**

Assigning qualified interpreters is considered one of the most effective approaches to facilitate communication in language-discordant encounters in mental healthcare. However, particularly in settings with fewer resources, they are not always available and informal practices are often used.

**Objective:**

This study aimed to investigate informal interpreting practices in mental healthcare in South Africa, focusing on security guards (SGs) serving as interpreters.

**Methods:**

Guided interviews were conducted with SGs (*n* = 12) and mental healthcare providers (MHCPs) (*n* = 18) at a psychiatric hospital in South Africa. The interviews were audio recorded, transcribed verbatim and analyzed using a thematic analysis approach.

**Results:**

Despite recognizing that SGs serving as interpreters is not an ideal solution to overcome language barriers and could potentially jeopardize the quality of treatment and its outcomes, MHCPs reported relying heavily on them due to the underrepresentation of South Africa’s linguistic diversity among them. Given the lack of formal interpreting services, the perceived racial, linguistic and socioeconomic similarities between SGs and some service users, as well as their immediate accessibility, were described as beneficial to providing a minimal level of care (e.g. obtaining information about service users’ backgrounds, getting an understanding of their symptoms, psychoeducation, explaining treatment options). Drawbacks reported are SGs being pulled away from their actual duties, experiencing emotional distress, juggling multiple sometimes conflicting roles, and the risk of incorrect interpretation, which could compromise ethical standards of care. Additionally, the complexity of power became apparent: While SGs hold little institutional power within the mental healthcare system, they become powerful figures when serving as interpreters.

**Conclusion:**

It can be assumed that MHCPs will resort to informal interpreting practices as long as effective alternatives are lacking. In doing so, risks such as reduced quality of care are accepted, and the consequences and effects on those serving as interpreters are neglected, which raises concerns from an ethical point of view.

**Supplementary Information:**

The online version contains supplementary material available at 10.1186/s12913-024-11722-5.

## Introduction

### Verbal communication and language barriers in mental healthcare services

Language discordance between service users and healthcare providers is a challenge which may compromise healthcare outcomes. This issue may be even more pronounced in mental healthcare, as symptoms are often not directly observable and effective verbal communication is even more critical [1]. Language-discordant communication can lead to a higher risk of misunderstandings, misdiagnosis, ineffective treatment, lower treatment satisfaction and poorer health outcomes than language-concordant encounters [2–4]. Since verbal communication is essential for building rapport and trust, language barriers can also negatively affect the therapeutic alliance [5]. Lack of fluency in the official or dominant language(s) of a region or country and its healthcare systems is recognized as one of the main global barriers to accessing healthcare services for individuals from linguistically diverse backgrounds [6–8].

### Qualified interpreters: the ideal vs. reality

Different practices exist globally in mental healthcare to facilitate verbal communication in language-discordant encounters. These include nonverbal communication, family members, multilingual personnel or other service users as interpreters, receptive multilingualism (interlocutors using different languages but still understanding each other), technological translation tools, or qualified (often called professional) interpreters [1, 9–16]. Although most studies emphasize that assigning qualified interpreters is the most effective approach to providing high-quality mental health care to linguistically diverse service users [1, 10], this ideal often remains unattainable in reality.

On the systemic and institutional level, providing qualified interpreting services is linked to a political and social attitude towards human rights, equity, inclusivity and non-discrimination, and the provision of resources [17, 18]. While the importance of language and the consequences of language barriers are increasingly emphasized in research and clinical practice, this is hardly reflected at the socio-political level in many countries [19, 20]. In most countries, there are no legal standards defining what characterizes a qualified or so-called professional interpreter or what their minimum qualifications should be [21]. Qualified or professional interpreters often comprise individuals with varying levels of training and certifications, ranging from a few hours of training to a graduate degree [17, 22]. Furthermore, there are usually no institutional guidelines regulating when an interpreter must be called in. Therefore, the presence of an interpreter in a language-discordant encounter usually depends on the initiative and knowledge of the mental healthcare provider (MHCP) [23] or established standards within an institution. Even when MHCPs are eager to include a qualified interpreter, none may be available or financial, personnel or time resources may be insufficient. Although using qualified interpreter services may prevent the escalation of long-term costs (through minimization of unnecessary repeat visits, for example), the initial financial costs are estimated to be higher and might be a burden [24, 25]. In many countries, the funding of interpreting costs is still largely unregulated, leading to uncertainty about who is supposed to cover the costs [22, 26]. The lack of resources and standards results in MHCPs and institutions continuously using ad hoc practices such as family members or untrained multilingual staff to overcome language barriers. This may seem more cost-effective and less time-consuming, but it could negatively impact the treatment process and outcome [11, 17, 27]. Most of what has been published about language discordance in mental healthcare comes from high-income countries [28]. These issues, however, are just as salient in low and middle-income countries, including South Africa [27], where this study is located.

### The South African context

South Africa is currently considered an upper-middle-income country [29]. With its past of colonialization and 12 official languages (including South African Sign Language), it emerges as a particularly interesting context in terms of language barriers in mental healthcare. In many parts of the world, colonial supremacy has led to the dominance of the languages of the colonizers at the expense of indigenous languages [30, 31], including South Africa. During apartheid, a system of institutionalized racial segregation and oppression that formally ended in 1994, only English and Afrikaans were considered as official languages and various indigenous languages were suppressed. The consequences of the past are still present in today’s mental healthcare system. While the majority of the population speaks isiZulu (24.4%) or isiXhosa (16.3%) inside households [32], the majority of MHCPs in higher positions, such as psychologists and psychiatrists, primarily speak English and have limited proficiency in local languages, apart from Afrikaans, despite measures to diversify the workforce, such as departmental and provincial policies for proficiency in other languages for newly hired MHCPs [20]. The linguistic disparities between MHCPs and service users demonstrate that even 30 years into democracy, access to high-quality mental healthcare remains deeply entwined with one’s racial and socioeconomic status [27, 33].

Despite the linguistic diversity of South Africa’s population and the disparities between languages spoken by MHCPs and service users, limited formal interpreting systems exist in mental healthcare settings [34]. Consequently, informal ad hoc practices often take place, including nurses, family members, cleaners or security guards (SGs) being used as informal interpreters to bridge the gap and facilitate verbal communication [11, 20, 27, 35]. Though some work has been done on the role of cleaners, family members, and nurses in South Africa, the role of SGs has not yet been explicitly explored [11, 20, 27, 35]. This study aimed to shed light on informal interpreting practices conducted by SGs in mental healthcare in the South African context, focusing on practices at a large psychiatric hospital.

## Materials and methods

### Research design

As part of a larger qualitative research project on the role of SGs, we conducted semi-structured interviews with SGs and MHCPs at a psychiatric hospital in the Western Cape of South Africa. Here, findings from a small sub-analysis focusing on SGs acting as informal interpreters are presented. The reporting of methods is in accordance with the consolidated criteria for reporting qualitative research (COREQ) [36].

### Setting

The study was conducted with SGs and MHCPs working at a psychiatric hospital in the Western Cape of South Africa. The hospital consists of different inpatient units, including forensic and acute ones. It provides mental health care assessments, crisis intervention and (short-term) treatment for adults with a broad range of psychiatric disorders. Most SGs working at the hospital are not directly employed by the hospital but by an external private security company and contracted to work in government hospitals. MHCPs are employed directly by the hospital.

### Participants and data collection

SGs were recruited in person following a purposive sampling approach [37]. SGs who worked at the psychiatric hospital with sufficient English, isiXhosa or isiZulu language proficiency were asked to participate. All SGs included in this study were employed by a private company and not directly by the hospital (outsourced). The aim was to select SGs of diverse genders, ages and work experiences to reach a maximum variation sample [37].

MHCPs were recruited in person, via email, or by phone following a purposive sampling approach [37]. Included in the study were MHCPs who were employed at the psychiatric hospital with sufficient English language proficiency. The aim was to select MHCPs who differ with respect to their profession, years of work experience, gender, and age [37].

Semi-structured guided interviews with SGs and MHCPs were conducted in person and one-on-one between October and November 2022. Two different interview guides for the SGs and MHCPs were developed following Helfferich’s SPSS approach of collecting, reviewing, sorting and finally subsuming questions formulated by the research team members [38]. SHR pilot tested both guides with research assistants to assess the feasibility and interview duration, resulting in no significant changes.

The interview guide was developed for this study but will be published elsewhere (Hanft-Robert S, Shongwe L, Cossie Q, Sithole P, Roos T, Mösko M, et al: The role of security guards in psychiatric care. A qualitative study with security guards and mental health care professionals in South Africa, forthcoming). The interviews with MHCPs covered their experiences with SGs acting as informal interpreters, the perceived challenges and benefits for themselves, the service users, SGs and the institution. Similarly, the interview guide for SGs covered their personal experiences, perceived challenges and benefits of acting as informal interpreters. Furthermore, a short questionnaire was administered to collect information on socio-demographics, work experience and SGs’ training. MHCPs were interviewed in English by SHS, a female M.Sc. psychologist. SGs were interviewed in isiXhosa and isiZulu by LSh, a female M.Sc. researcher. There was no previous relationship between the interviewers and the interviewees. All interviews were digitally audio-recorded. The interviews conducted in English were professionally transcribed verbatim. The interviews with SG speaking isiXhosa or isiZulu were transcribed verbatim and translated into English by a research associate. SHR and LSh proofread all transcripts. Any personal data that could potentially lead to the identification of interviewees (e.g., participant names, employers, institutions) was either deleted or altered. Transcripts were not returned to participants.

### Data analysis

Data analysis was conducted collaboratively by SHR and LSh, in close coordination with LSw and QC, employing the Thematic Analysis (TA) approach by Braun and Clarke [39]. All authors have experience with qualitative research projects and conducting and analysing interviews. Braun and Clarke [39] provide a comprehensive six-phase guide that includes familiarization with the data, coding, searching for themes, reviewing, defining and naming themes, and writing up. The coding process was primarily inductive, meaning the categories were formulated based on the interview data. The interviews with the SGs and the MHCPs were analyzed as one set. The main and sub-themes were created jointly across both groups. It was then checked whether themes were mentioned in both or just by one group. Since most themes were mentioned by both groups, we decided to present the results from both groups together but indicated by whom the themes were mentioned. Intersubjective comprehensibility and credibility [40] were ensured by SHR and LSh independently analyzing and discussing four interviews of MHCPs and all interviews of SGs. SHS analyzed the remaining interviews with MHCPs. Additionally, the final category system was critically reviewed and discussed within an interdisciplinary research team to guarantee intersubjective reproducibility and comprehensibility [40]. Study participants did not provide feedback on the results. Data was analyzed using MAXQDA 2020.

### Ethical considerations

Before the interview started, all interviewees provided oral and written informed consent to participate in the interview, to have the interview recorded, and to allow for the subsequent processing of the anonymized interview data for scientific purposes. Participation in the study was voluntary and not remunerated. The research was performed in accordance with the Declaration of Helsinki and has been approved by the Ethics Committee of Stellenbosch University, South Africa (Ethics reference number: SBE-2022-25557).

### Sample

In total, *n* = 18 MHCPs from different professions participated in this study. Among the MHCPs, *n* = 11 identified as female and *n* = 7 as male. Their mean age was 43 years (SD = 12.07), and their mean work experience in their respective profession was 16.81 years (SD = 12.16). Included were social workers (*n* = 2), nurses (*n* = 3), psychiatry registrars (trainee specialist, *n* = 2), doctors (*n* = 2), occupational therapists (*n* = 2), (clinical) psychologists (*n* = 4), and (forensic) psychiatrists (*n* = 3). All MHCPs reported having contact with SGs daily or weekly. Two said that they have received some kind of training on the role and work of SGs in that specific psychiatric hospital.

In addition, *n* = 12 SGs were included in the study. Among them, *n* = 8 identified as male and *n* = 4 as female. Their mean age was 39.08 years (SD = 10.51), and their mean professional experience was 5.92 years (SD = 7.94). SGs from forensic and acute units were included.

## Results

The following four main themes were identified, including several sub-themes (Table 1):

**Table 1:** Identified main and sub-themes

Main theme	Sub-themes
The healthcare system’s reliance on SGs serving as informal interpreters	1. South Africa’s linguistic diversity is often not represented among MHCPs 2. Lack of formal interpreting services
Benefits and challenges of SGs acting as informal interpreters	1. Perceived racial, linguistic and socioeconomic similarity between SGs and some service users 2. Immediate access 3. SGs being pulled away from their actual duties 4. Emotional distress for SGs 5. Lack of training and incorrect interpretation 6. Loss of therapeutically relevant information 7. Confidentiality concerns
SGs’ (expected but conflicting) roles while interpreting	1. Dual role of ensuring safety while interpreting 2. SGs as a valuable source of information 3. Service users’ confidant 4. Co-MHCP
Power dynamics surrounding interpreter-mediated encounters	1. Lack of recognition 2. The impact of status differentials

### The healthcare system’s reliance on SGs serving as informal interpreters

#### South Africa’s linguistic diversity is often not represented among MHCPs

While English was described as the most commonly used language by MHCPs, a significant proportion of service users primarily speak another language. Consequently, in order to facilitate communication in language-discordant encounters, both groups reported that SGs were frequently asked to provide interpreting services: *‘Yes*,* it helps both of them because you find that the doctor cannot understand Xhosa and the patient does not understand English*,* so now they both get helped.’* (SG03).

Some MHCPs voiced the concerns that their own language skills were insufficient in meeting the linguistic diversity of service users adequately: *‘So I speak Afrikaans*,* I speak English*,* and I speak a fair level of Xhosa to build rapport and show empathy. But I’m not going to be able to engage in a conversation that’s going to lead into three or four paragraphs.’* (MHCP01). In addition, some participants from both groups critically reflected on South Africa’s colonial past and the apartheid era, along with its lasting effects on the current healthcare system, which encompasses institutionally rooted racial and linguistic disparities between MHCPs and service users. One MHCP expressed that there is still reluctance on the part of the MHCPs to reflect and overcome such differences: ‘*But I think also that just speaks to us as a people*,* I mean*,* why don’t we speak each other’s languages? I think that is just poor. You know*,* it would be like if I lived in Germany for 25 years and didn’t learn a word of German and then complained every time someone said something in German. It would be bizarre*,* right? It is once again that colonial mindset of ‘I don’t have to change*,* I don’t have to do anything*,* I don’t have to be interested in this nation’. So someone else must do it. And even then*,* the idea that a professional would call a security guard to translate relates to the disrespect and the disdain that they have for the patient*,* the disdain they have for the security officer because nobody has asked the security officer whether he wants to listen to that.’* (MHCP14).

#### Lack of formal interpreting services

Due to a lack of formal interpreting services available for psychiatric institutions, almost all MHCPs stated that people who are not qualified as interpreters but speak service users’ language, such as relatives, nurses, cleaners or SGs, were asked on a regular basis to act as interpreters in language-discordant encounters. This was perceived as the only way to be able to communicate with service users: *‘Sometimes in the isiXhosa speaking groups where there’s a language barrier*,* if there isn’t a nurse available and they don’t understand something that’s been said or asked*,* then the patient might say to them [the SG] in their mother tongue*,* and they will translate for me. So that would be informal assistance that they give us.’* (MHCP16).

Nonetheless, some MHCPs pointed out that limited formal interpreting services do exist. However, they are usually external and either not known, or the commissioning process is overly complex and time-consuming and does not fit the dynamic reality of psychiatric services: ‘*It’s more often used*,* I think*,* with psychometric assessment. And even then*,* I still don’t think*,* if I had to pull the stats now and ask how many times have we used the translators*,* […] I’m not sure that we’re accessing them to their full potential.‘* (MHCP11).

### Benefits and challenges of SGs acting as informal interpreters

#### Perceived racial, linguistic and socioeconomic similarity between SGs and some service users

SGs working at the psychiatric hospital where the current study was conducted were perceived by the some MHCPs to be similar to some service users regarding their racial, linguistic and socioeconomic background. This perceived similarity proved convenient for them to serve as informal interpreters when needed, as explained by one MHCP: *‘And sometimes it’s also because of language. Because a lot of security guards (...) they speak Xhosa. And patients understand that*,* so they get along with these patients in their home language*,* which is also helpful to kind of get information.‘* (MHCP09).

#### Immediate access

Compared to formal interpreting services, some MHCPs preferred the use of SGs as interpreters due to the time constraints and immediate and convenient access: *‘I think only later did our department make interpreters available. But because it’s such a tedious process also to pick up the phone and make a booking*,* often you use what is on hand*,* and you don’t call upon these people. Sometimes with us*,* also families just pitch. It’s an opportunity you don’t want to miss*,* especially when you have to get collateral and you’ve been trying to trace family and suddenly they arrive. There’s no way you can pick up the phone and ask for an interpreter to come now. So you make of course use of whoever is there to interpret for you. So I think those are some of the challenges that we face.‘* (MHCP08).

#### SGs being pulled away from their actual duties

SGs stated that acting as an interpreter is an additional duty, not part of their core responsibilities. Similarly, MHCPs expressed concerns about utilizing SGs as interpreters because it hinders them from fulfilling their primary responsibilities and creates a gap somewhere else: *‘I just wonder what happens to the rest of the ward then if we are using the Security as an interpreter*,* then we sometimes lose the Security for the rest of the ward.’* (MHCP04).

Some MHCPs wondered if acting as interpreters might make SGs feel more valued and part of the team, increase their job satisfaction and perceived responsibility: *‘I would hope that maybe it would make them feel like they’re contributing more positively and that the medical team actually also needs them. So I think from an inclusivity perspective and feeling part of the team…in terms of job satisfaction and job reward*,* and a person just sitting on a chair and watching patients go up-and-down in the courtyard*,* actually being able to have a constructive role and they feel like*,* well*,* actually*,* doctors need me*,* the nurses need me*,* the OTs need me. I would hope that that would make them feel like they have a bigger and more important role.’* (MHCP12). However, most SGs reported that they perceive serving as an interpreter merely as being helpful and supporting the MHCPs in their work: *‘It’s just to help*,* otherwise*,* it’s not our job. […] No*,* there’s nothing I benefit.’* (SG03).

#### Emotional distress for SGs

Both MHCPs and SGs described interpreting as emotionally distressing for SGs: *‘But I think for the security guard it won’t be okay because our patients experience a lot of trauma and no one knows how then that could trigger the specific security guard into bringing up their own traumas. They have to sit in there and tolerate whatever conversation is happening*,* and they have to be part of this literally now because they are responding in their own language to what the patient is saying. So it’s like they are talking about themselves as well.’* (MHCP05). This could be particularly difficult to manage because SGs usually lack training and knowledge in mental health: *‘I think for the security guard if you think no training on mental health*,* often the material that is shared is extremely traumatic and I would be concerned about exposing someone to trauma vicariously if we haven’t done any work to prepare them.’* (MHCP11).

Many SGs expressed anxiety about their own competence to interpret, especially when the structure of the language used by service users was challenging: *‘You find that you don’t know what to take and what not to take; like*,* what am I going to say! [.] They [the MHCP] think I am not doing well…!’* (SG02).

While MHCPs had access to supervision or psychosocial support services, such offers are unavailable for SGs: *‘And like I said*,* as government workers we have an outlet. You can speak to your colleague. You can phone up a counsellor*,* and access a counsellor immediately. You can go to a counsellor or a counsellor can come here. They can do a debriefing. But as a security guard*,* you can’t.’* (MHCP08).

#### Lack of training and incorrect interpretation

All MHCPs expressed concern about incorrect interpretation when using SGs as interpreters. SGs usually lack mental health knowledge and are not familiar with specific mental health terms: *‘But having them as an interpreter can be a drawback also because obviously*,* they don’t have any medical training*,* they don’t know any medical terms and I know in some of the languages*,* some of the words might have a different meaning altogether when they tell the patients and it might be interpreted completely in a different way and you would get eventually the wrong assessment of the patient. So I don’t think that is their role…they shouldn’t be taking on that additional role. But … I do know sometimes I do know people have asked them.’* (MHCP03). Thus, MHCPs expressed their preference of having another professional with a mental health background, e.g. nurses, to assist with interpretation: *‘But sometimes we have…I’ve told you that I don’t on principle ask security guards to interpret for me. I’ll always ask a nurse.*’ (MHCP17).

SGs’ lack of mental health knowledge could result in abbreviated, summarized and incomplete interpretations. Especially when the service user experiences delusions or uses vulgar language, MHCPs experience SGs struggling with providing complete and correct interpretation. SGs added that they tend to make sense of what service users say when conveying it to the MHCP. However, sometimes they do not understand that particularly what the service users say and how they say it is crucial to the diagnosis and treatment process: *‘There’s only one problem; you find that the patient is answering something that is off-topic*,* now you have to structure it the way that the patient*,* without rectifying it*,* and you find that something you cannot say to them*,* because these people are not well.’* (SG03). Additionally, MHCPs stressed the importance of linguistic nuances in mental healthcare: *‘Because for me it is fascinating that in mental health we cannot always see what is wrong with the patient. We’d need them to tell us how they experience their symptomatology. So*,* we look for nuance. We look for details. And so if someone is not trained to catch the nuance and to catch the detail*,* then we don’t get a full picture.‘* (MHCP18).

Additionally, some SGs revealed that they sometimes lack sufficient language skills for interpreting properly, which could have a negative impact on their interpreting service: *‘But the doctor has asked me*,* they asked if I could try and make them understand*,* and I tried. I also don’t know English well*,* but I tried*,* I tried and pushed through […]. I can understand*,* but sometimes I realize that I am just guessing’* (SG04).

#### Loss of therapeutically relevant information

The incorrect interpretation could result in a loss of therapeutically relevant information about the service user, as feared by almost all MHCPs: *‘So my worry would be that again when we’re interviewing someone*,* we are obviously looking for clinical signs*,* so because I don’t know what’s getting lost in translation*,* how much of what is related to the patient are my words versus the security guard’s interpretation on them and what do they choose to relay back. I think I’m worried that important facts aren’t relayed back because it’s assumed that it’s irrelevant or not useful. Because they are not aware of clear signs of symptoms or they don’t understand why we are asking something that we’re asking*,* so I think that that’s one massive concern just for a clinical understanding and a medical knowledge perspective.’* (MHCP12).

#### Confidentiality concerns

The presence of SGs could make the service user more cautious about speaking openly due to concerns about their professionalism and confidentiality as reported by MHCPs: *‘Something for me*,* of course*,* it does impact on the information the patient is going to give you. I think it really hampers because you don’t know it for sure*,* but you can pick up on the milieu. You can pick up on the patient’s anxiety. I mean*,* a lot of the information*,* as I’ve said*,* is confidential. In the forensic unit*,* sometimes it’s horrific crimes you have to relive*,* you have to retell it. Or*,* say*,* in a therapy session where patients have to really grapple and face what it is they’ve done and the impact on their families*,* and impact on the community. So you sometimes feel that patients are not fully participating in that process.’* (MHCP10).

It could also be unsettling for service users that the same SG, who has heard personal things about the service user, will also be around them in the ward: *‘Ja*,* I mean*,* it’s obviously unsettling for the patient because the patient knows that the security guard is going to be in their environment and now the security guard knows something about them that maybe they don’t want the security guard to know.’* (MHCP17). Another MHCP added that service users might be afraid of getting stigmatized by SGs: ‘*And then also*,* the difficulty would then come with a stigma*,* you know with the patient feeling that a security guard who is now sitting with them in the ward*,* amongst other patients might be stigmatizing them because of what they know about them.’* (MHCP04).

### SGs’ (expected but conflicting) roles while interpreting

#### Dual role of ensuring safety while interpreting

It was described by both groups that SGs acting as interpreters were also expected to perform their duties as SGs and ensure safety in the room: *‘So focusing on them on what their primary task is*,* because they often get more into an interpreter role and then not observing other*,* I don’t know what the word*,* hazards*,* or dangerous situations*,* so they could then lose that role as a security person. […] Also you feel safer in the situation because you know there is a security present*,* so automatically you feel calmer; the patient responds to that calmness; it’s just a more pleasant interview.’* (MHCP04).

It was described by MHCPs that service users might see SGs primarily as individuals responsible for ward safety, which could be an obstacle to establishing rapport: *‘Absolutely! Because the security guard has a dual role there. When they go outside*,* the security guard is going to tell them what to do and what not to do. And then*,* in the room*,* where you want to create a safe space for the client*,* I don’t know how that dual role of a security guard can be because interpretation in the room versus their role outside the room is quite different. […] And that could impact on the rapport you build with the client*,* or how safe they feel in the room with the client*,* for example.’* (MHCP13). Past negative encounters between service users and SGs could contribute to feelings of mistrust and reluctance on the part of the service users: *‘Because*,* like I say*,* they might*,* you know*,* the patient has had a negative experience with a security guard in the past*,* or you know*,* they view security guards as*,* you know as*,* this is my enemy*,* kind of*,* you know*,* so they might not want to talk to them.’* (MHCP03). In general, service users might perceive SGs as figures of authority who have the capacity to use force, which could affect the therapeutic process: *‘I just mean*,* for example*,* if there’s something that perhaps reminds the patient of the abuser or the perpetrator*,* that may lead to them becoming more withdrawn and so are they not going to want to be forthcoming with information. And perhaps if you remove that person*,* they suddenly will reveal it to you.’* (MHCP07). Furthermore, one MHCP emphasized that the dual role of SGs within the MHCP-service user interaction could place the SG in a potentially conflicted situation. Because they bear the responsibility for ensuring safety, they might feel hesitant to interpret something that could potentially trigger aggressive behaviour in the service user: *‘Because I suppose the highest point of risk for them is when they are with a staff member*,* that if they become violent or aggressive*,* they might have to intervene. So they don’t want to say anything that might be triggering*,* which an interview might do from time to time to test certain reactions or defence mechanisms or something like that.’* (MHCP10).

#### SGs as a valuable source of information

SGs serving as interpreters were also perceived as a valuable source of information for MHCPs. MHCPs described that they have received important information about service users and their current mental state through the SGs, who are always around them at the wards. Sometimes SGs and service users came even from the same communities, which was perceived as beneficial for getting background information: *‘They also sometimes come from the same community setting*,* so they already have a performed*,* or they know someone who’s in the church with this patient or lives in the same area*,* or they understand the gangs involved*,* or they understand the dynamics in that community better than we do understand it. So they can relate to the patients a bit more.’* (MHCP04).

#### Service users’ confidant

Since SGs spend a lot of time with service users at the ward, sometimes share a racial, linguistic, cultural and socioeconomic background or come from the same community, it was reported by both groups that service users might relate more to SGs, and both form a strong personal bond. This bond could result in the SG being the service users’ confidant, providing a safe space in the interpreter-mediated encounter: *‘Like*,* maybe you find that the doctor didn’t know that the patient isn’t able to*,* you see? So*,* they just asked me*,* ‘Because you speak isiXhosa*,* can’t you help me?’ […] Yeah*,* to adjust. But in my case*,* at least I can understand. And I try for my patient to*,* yeah*,* to be alright.’* (SG02). The presence of SGs as interpreters was described as helpful for service users to open up and for MHCPs to build rapport with service users, as reported by some MHCPs: *‘And then because he had a good relationship with the patient*,* the patient was willing to engage as long as he [the SG] was in the room constantly. […] So it just opens channels of communication.’* (MHCP04). However, this could lead to meshed relationships and blurred boundaries between SGs and service users as described by both groups: *‘The pros obviously understand well*,* understand what sets you off*,* understand your ins-and-outs*,* but the adverse is that there are also negative connotations that are attached where I feel like I can speak to you the way I want to or I feel I can treat you the way I want to. That’s the negative.’* (MHCP01).

#### Co-MHCP

During the interviews, it became evident that despite a lack of mental health knowledge and training, SGs acting as interpreters are seen as a crucial part of the mental health assessment of service users and might be pushed into the role of a co-MHCP as described by one MHCP: *‘So I asked the translator*,* and that’s the example where I said that the security just said*,* no*,* he’s not right. And I was like*,* we need to try and delve a bit further. And then he said that no*,* when he asks questions he doesn’t reply appropriately and he talks off points. And then that helped a bit more in terms of saying he’s psychotic because his thoughts are disordered. Then*,* he also had delusions. I mean*,* because it makes it a bit difficult to assess someone just based on the effect. So that was the one case.’* (MHCP07).

### Power dynamics surrounding interpreter-mediated encounters

#### Lack of recognition

It was described by MHCPs that SGs usually have a lower educational status and come from lower socioeconomic backgrounds, often without any formal training for working as SGs. This results in SGs being frequently overlooked and lacking respect and recognition from both MHCPs and service users: ‘*So there is no job satisfaction. There is no day where you go home*,* and you feel like I did good work today. Because the whole day*,* either the patients are telling you all kinds of expletives about who you are*,* or the nursing staff are complaining about the fact that you are not responsive and you’re not doing what you’re supposed to do. Or*,* you’re getting feedback from your line managers that the hospital managers complain that the security staff are not behaving appropriately. It’s a no-win scenario. Whichever way you cut it*,* you are not going to come out a winner. You’re not going to come out feeling like*,* okay*,* I’m valued*,* I matter. Because at that level*,* you are expendable*,* and because of the unemployment rate in this country*,* that position is filled within minutes.‘* (MHCP14).

Participants from both groups expressed that despite SGs being an important part of psychiatric hospitals, they are not considered integral members of the professional team: *‘Because when you are the security guard sometimes in some places you are looked down upon as though you are someone insignificant; when someone wants something from you*,* or they want to come in*,* and I say; ‘Sister*,* you are not allowed to enter here!’’* (SG05).

The lack of professional recognition results in the perception of SGs being blamed easily by MHCPs or service users. SGs are often targeted and seen as scapegoats, particularly by service users. One SG described: “*Here*,* there’s just one thing I don’t like- most of the time*,* whenever an incident occurs*,* they make it as if it is the security guard’s fault.”* (SG11). And one MHCP added: *‘I think that they are kind of overlooked*,* and so their opinion or their kind of say doesn’t mean much in an argument. The patient always kind of*,* what makes it even more difficult is that if it’s a revolving ward patient*,* that knows how that ward works*,* so they are looking for a scapegoat*,* there is that person to blame in the Security.’* (MHCP9).

Given that most SGs frequently rotate through various wards rather than being permanently assigned to one, MHCPs described it as not possible or worth it to invest in forming personal relationships with SGs: ‘*And then six months later*,* we had somebody new. And I realized you will never really form a connection.’* (MHCP11).

#### The impact of status differentials

The MHCPs are perceived by SGs as having a higher status, and their requests, including being asked to provide interpretation, may not be refused. SGs may perceive themselves as not suitable for acting as interpreters, e.g., because their own English language skills are insufficient or because they feel overwhelmed with interpreting for individuals who are mentally not well. One MHCP reflected on their privileged position within the mental healthcare system and possible power dynamics between them and SGs: *‘So even though South Africa has decolonized itself from a political aspect*,* they haven’t really done that in terms of institutionally. So because of the virtue of the position that I occupy*,* I have a top-down kind of power where I can exercise a disproportionate amount of force*,* not physically*,* but just through the words I say and through the things…my actions. And so there’s still the sense of disempowerment […]. And then they come into a workspace and here I am with my privileged voice saying*,* you are not doing this and you are not doing that*,* you should be doing this. So they are frustrated. They are not protected by their own organization. They are not protected by our members of staff. They are not protected by the patients. So they are on their own. So a lot of the time they are working in unfairly distressing conditions. That obviously does need some reflection and pause for thought.’* (MHCP14).

Participants from both groups expressed SGs’ precarious working conditions, and lack of job security lead to a constant fear of being replaced or fired, which contributes to SGs’ compliance with MHCPs’ demands: *‘And that whatever is asked of them*,* they just comply and do. Also*,* out of fear of getting bad reports*,* of being moved out of the area or possibly being out of a job. So I think they are also in a very vulnerable position where*,* as I’ve said*,* they often comply.’* (MHCP8).

Furthermore, one MHCP shared the historical context of mental health institutions. During apartheid, the policy of all government institutions, including the hospital where the study was conducted, was to segregate White and Black individuals in their treatment. Consequently, SGs, who are predominantly Black, still encounter the challenge of working in an environment that was previously characterized by high levels of institutionalized racial segregation and discrimination: *‘Whereas this environment is a reminder for everybody of their own suffering […]. So I think that there is something about this facility that also triggers something in the staff.’* (MHCP11). It was reported by both groups that SGs often find themselves mediating between a White MHCP and a Black service user: *‘Yes*,* it has happened. Well*,* let me not say it has happened; it happens…you can translate for them. For example*,* maybe the doctor is White*,* then the patient is Black*,* so now you have to; the patient does not understand English*,* […] so I translate.’* (SG10).

## Discussion

This qualitative study explored informal interpreting practices conducted by SGs in a psychiatric hospital in the Western Cape of South Africa. The perspectives and experiences of MHCPs and SGs were assessed through qualitative interviews. Four main themes emerged comprising several subthemes: (1) the healthcare system’s reliance on SGs serving as informal interpreters, (2) benefits and challenges of SGs acting as informal interpreters, (3) SGs’ (expected but conflicting) roles while interpreting, and (4) power dynamics surrounding interpreter-mediated encounters.

### Is a little communication better than no communication?

Participants in this study reported that most MHCPs speak predominately English and that their language skills were often insufficient to communicate effectively with service users from linguistically diverse backgrounds. This is in line with previous findings and emphasizes the historically rooted racial and linguistic disparities between MHCPs and service users in South Africa’s healthcare system [27, 33, 41]. Although there are departmental and provincial policies for language proficiency for newly hired MHCPs, such as the requirement to speak two of the three main languages of the province, the language profile of MHCPs in the hospital does not appear to have changed, which raises the question of whether these guidelines are being adhered to. Although participants in the present study stressed that interpreting is a profession that requires special skills and competencies [17, 42], a lack of formal interpreting services and qualified interpreters was expressed. This created a challenging dilemma for MHCPs: they were caught between a desire to offer high-quality care and the practical need for immediate communication and quickly accessible solutions. Additionally, SGs were valued as a convenient and easily accessible solution to bridge the language gap due to their perceived racial and linguistic similarities with some service users. However, to avoid racial bias, it should be noted that people cannot be perceived as a homogenous group simply because they share racial, linguistic and socioeconomic similarities or come from the same community. There might also be great diversity within these groups, e.g., regarding language proficiency or cultural beliefs.

The reliance on SGs as informal interpreters, while far from an ideal solution, was described as necessary in a system with a great diversity of languages but lacking or not easily accessible formal interpreting services. Similar findings have been found in other countries. For example, Keller and Carrascoza-Bolanos (2023) reported that healthcare professionals often resort to using informal interpreters in Southern California due to time constraints, heavy workloads and urgent service user needs [43]. In this study, MHCPs acknowledged that this reliance on SGs may compromise the quality of care. SGs lack the necessary training and expertise in interpreting and mental health; some may even have inadequate English language skills. This is in line with previous studies on informal interpreting practices reporting that lacking interpreting and language competencies could lead to a loss of information, misunderstandings, frustration or even misdiagnosis [11, 41, 44–46]. Hagan et al. (2020) noted that untrained interpreters might “clean up” what service users are saying, potentially leading to the unintentional omission of symptoms or other relevant information [41]. Similarly, MHCPs in this study expressed the importance of nuances and how things are said in mental healthcare, which might get lost when SGs serve as interpreters. However, due to the urgency of providing care and the absence of better alternatives, SGs were often perceived as the only solution to establish at least some sort of verbal communication with service users. Previous studies concluded that the utilization of informal interpreters, such as SGs or cleaners, on the one hand, potentially compromises ethical standards of care. On the other hand, they emerged as the sole viable option for verbal communication and were thus regarded as beneficial for providing minimal care to service users [11, 41].

### Security guards’ multiple roles while interpreting

The primary role and responsibility of SGs in a psychiatric hospital is to maintain safety for both the staff and the service users [47]. The present study revealed that SGs are expected to shoulder the dual responsibility of interpreting while simultaneously ensuring safety. While MHCPs perceived this role duality as beneficial and reassuring, especially when interacting with agitated or aggressive service users, balancing these two crucial tasks at the same time can be challenging and conflicting for SGs. It was shown that SGs’ primary responsibility for ensuring safety could influence their interpretation service, for instance, leading to hesitation to convey information that could potentially provoke aggression or violence. Both formal SG and informal interpreting roles may be compromised by role confusion. Despite the clear benefits of multiple roles for the current provision of services, this complexity is a major challenge.

In addition, it was described that SGs might be perceived as figures of authority who lack trustworthiness in the eyes of service users. Prior negative interactions between SGs and service users may foster feelings of mistrust and reluctance on the part of the service user to share sensitive information and impede the rapport and the development of the therapeutic alliance with the MHCP.

Due to their continuous presence at the wards, SGs might become familiar with service users and form a strong personal bond over time. While this was described as helpful for the service user to open up and for MHCPs to facilitate trust and rapport, it also led to meshed relationships and blurred boundaries in the interpreter-mediated encounter. This complements previous findings demonstrating that cleaners acting as informal interpreters fail to maintain a consistent focus on their role as interpreters [44] and tend to align with either the MHCPs or the service user, thus creating a clinical situation characterized by asymmetrical relationships [11, 48]. Moreover, it was shown that cleaners serving as informal interpreters may use their prior observations and knowledge of service users to convey information to the MHCPs, which goes beyond merely interpreting what has been said in the specific situation [11]. These practices raise ethical concerns regarding service users’ privacy and confidentiality, as information might be shared without their consent.

### Power dynamics outside and within an interpreter-mediated encounter

In the present study, the profession of SGs was found to be generally undervalued and received little recognition, largely because it typically does not require formal professional training or certification. SGs described being overlooked and lacking respect and acknowledgement from both MHCPs and service users. Although SGs’ vital role in the healthcare system became evident in this study, they were not perceived as equal team members at the psychiatric hospital where the study was conducted. The role of SGs in psychiatric hospitals and their precarious working conditions are described in more detail elsewhere (Hanft-Robert S, Shongwe L, Cossie Q, Sithole P, Roos T, Mösko M, et al: The role of security guards in psychiatric care. A qualitative study with security guards and mental health care professionals in South Africa, forthcoming). MHCPs were commonly perceived as holding superior positions with higher status within the healthcare system. Consequently, SGs often found themselves in a precarious situation where they were reluctant to decline requests made by MHCPs, such as serving as an interpreter, even if they felt unqualified. The fear of potential repercussions, such as being fired, often compelled SGs to comply with these demands. In addition, racial differences between SGs, MHCPs and service users might exacerbate such power disparities. In line with findings by Smith et al. (2013), some SGs described that taking on the role of an interpreter made them feel more valued and integrated into the team, which in turn could increase their job satisfaction and sense of fulfilment [11]. However, the majority perceived it as additional work and experienced interpreting as emotionally distressing due to being exposed to service users’ traumatic experiences while lacking training in mental health and psychosocial support or supervision. In addition, it might be possible that the pressure of fulfilling an additional role which is not part of their job description can be distressing for SGs.

Though SGs hold little formal institutional power, they may gain more informal power when serving as interpreters since both MHCPs and service users rely on their language skills. Mason and Ren (2012) elaborated that while lacking institutional power, interpreters are often equipped with power within the encounter due to their bilingual and bicultural expertise [49]. Rudvin (2005) noted that the person who interprets is the only person who can understand what all interlocutors are saying at all times, which can be considered an enormous source of power [50]. Regarding SGs acting as informal interpreters, the power dynamics might be especially crucial. SGs are usually perceived as powerful figures or authorities. At the same time, they lack professional recognition for their work, are often overlooked and have little institutional power within the mental healthcare system. This changes when they are used as interpreters and find themselves in a powerful position because of their language expertise. An in-depth analysis of the power dynamics surrounding SGs serving as interpreters would be interesting for future research.

### Strengths and limitations of the study

This study was conducted in just one psychiatric hospital in South Africa. Therefore, the results may be context-specific and it is not clear how generalizable they are. To increase the variation in results, it would be useful to include multiple psychiatric hospitals in different regions in future studies. The qualitative approach allowed for an in-depth exploration of the complex dynamics surrounding SGs serving as interpreters. Despite the relatively large sample size for qualitative studies, data saturation cannot be ensured. Due to time constraints, this study could not conduct further interviews. In future studies, as many interviews as possible should be conducted until data saturation is reached. In addition, the interviews were conducted by two different interviewers, which may lead to differences in how the interviews were conducted. However, both interviewers had a high level of expertise in qualitative research and conducting interviews; the interview guide was developed jointly and served as an orientation during the interviews. Moreover, the two interviewers made it possible to include people who speak English, isiXhosa and isiZulu. The heterogeneity of the sample and the inclusion of different MHCPs, such as psychologists, doctors, nurses and social workers, as well as SGs working at different wards in the psychiatric hospital, can be considered the greatest strengths of the study. While our study has already explored the two perspectives of MHCPs and SGs, it would be useful for future studies to also include service users’ perspectives. The study results provide in-depth and comprehensive insights, and the themes identified can serve as a database for further quantitative studies.

## Conclusion and implications

Despite recognizing this is not an ideal solution, the reliance on SGs serving as interpreters by MHCPs reflects the lack of effective alternatives to address language barriers in South Africa’s mental healthcare system. It can be assumed that informal interpreting practices will be continuously used due to the absence of better options. Thus, institutions should offer training and organizational support to enable effective communication between MHCPs and service users in all situations [51]. Based on the study results, some recommendations can be made for this practice. For instance, SGs acting as interpreters should be asked for their consent before assigning them as interpreters. The service users should also be asked for their consent. It should be ensured that serving as an interpreter does not interfere with their primary duties. Additionally, they should be informed about the situation they will be in and educated about mental health. Given the potential psychological distress that interpreting can cause, a debriefing session should be conducted afterwards. MHCPs should know the risks associated with SGs serving as interpreters, e.g., information loss due to limited language skills. The acceptance of risks and neglect of the impact on the individuals serving as interpreters (e.g. emotional distress) raises ethical concerns and highlights the urgent need for the development and implementation of effective, ethically appropriate alternatives in mental healthcare systems to ensure that the quality of care remains uncompromised despite language barriers.

## Supplementary Information

Supplementary Material 1.

## Data Availability

No datasets were generated or analysed during the current study.

## Abbreviations

SG: Security guard
MHCP: Mental health care provider
